# Case Report: Mild BRIC-like cholestasis despite a gross *USP53* deletion—novel findings and literature review

**DOI:** 10.3389/fgene.2025.1670664

**Published:** 2025-11-21

**Authors:** Ekaterina Nuzhnaya, Tatiana Cherevatova, Ekaterina Lotnik, Aleksandra Shagiazdanova, Zhanna Markova, Ekaterina Filimonova, Olga Parshina, Anastasiia Buianova, Anastasia Bobreshova, Andrey Marakhonov, Natalia Semenova

**Affiliations:** 1 Research Centre for Medical Genetics, Moscow, Russian Federation, Moscow, Russia; 2 Russian Children’s Clinical Hospital, A Branch of Pirogov Russian National Research Medical University, Moscow, Russia; 3 Institute of Translational Medicine, Pirogov Russian National Research Medical University, Moscow, Russia

**Keywords:** Usp53, BRIC, PFIC7, low-GGT cholestasis, pediatric hepatology, gross deletion, compound heterozygous variants

## Abstract

We report a pediatric case of cholestatic liver disease associated with two novel compound heterozygous variants in the *USP53* gene: a truncating c.1219A>T (p.Lys407*) variant inherited from the father and a maternally inherited gross deletion involving exons 13–19. Despite the disruptive nature of these variants, the patient presented with a benign recurrent intrahepatic cholestasis (BRIC) characterized by episodic pruritus, jaundice and elevated bile acids with preserved liver function between episodes. Liver histology revealed fibrosis with a cholestatic component, consistent with mild progressive familial intrahepatic cholestasis (PFIC) features. Molecular diagnosis was confirmed by whole-exome sequencing (WES), chromosomal microarray, and Sanger sequencing. A systematic review of 39 published cases was conducted, revealing that *USP53*-related disease exhibits broad clinical variability, ranging from BRIC to PFIC7. Our findings expand the spectrum of *USP53* variants, underscore the relevance of large deletions and emphasize the inclusion of *USP53* in genetic panels for idiopathic low-gamma-glutamyl transferase (GGT) cholestasis.

## Introduction

PFIC is a rare group of autosomal recessive liver diseases characterized by early-onset cholestasis, pruritus, and progressive liver dysfunction, often leading to transplantation (Alam et al., 2024; Zheng et al., 2023). Recently, biallelic variants in *USP53* have been identified as a cause of PFIC7, expanding the spectrum of cholestatic disorders (Maddirevula et al., 2019).

Although *USP53* encodes a catalytically inactive deubiquitinase, it serves an essential structural role in maintaining the integrity of tight junctions through its interactions with TJP1 (ZO-1) and TJP2 (ZO-2). Both TJP1 and TJP2 are scaffolding proteins that organize and stabilize tight junction complexes by linking transmembrane components such as claudins and occludins to the actin cytoskeleton, thereby regulating paracellular permeability and epithelial polarity. Disruption of USP53 interferes with its association with these proteins, leading to defective tight junction assembly, weakening of the canalicular membranes in hepatocytes, and subsequent cholestasis. In addition, because tight junctions formed by TJP1 and TJP2 are also critical for maintaining ionic homeostasis and barrier integrity in cochlear epithelial cells, USP53 dysfunction can impair cochlear tight junctions, providing a mechanistic explanation for the sensorineural hearing loss observed in affected individuals (Xia et al., 2024).

Clinically, *USP53*-related disease often presents with a milder, benign recurrent intrahepatic cholestasis (BRIC)-like phenotype, featuring episodic jaundice and pruritus with slow disease progression. Hearing loss has been inconsistently reported (Alhebbi et al., 2020; Zhang et al., 2020).

To date, only two patients have been described with сopy number variation (CNV) in *USP53* (Bull et al., 2020; Cheema et al., 2023). Here, we report a unique case of a patient with BRIC-like presentation carrying a novel nonsense variant and the largest reported *USP53* deletion (exons 13–19). We also review previously reported PFIC7 cases to highlight the phenotypic variability and diagnostic importance of *USP53* variants.

## Case presentation

A 16-year-old male patient was referred from the Department of Gastroenterology at the Russian Children’s Clinical Hospital with complaints of pruritus and abnormal blood test results.

He is the only child of his parents’ current marriage. His mother has a healthy son from a previous marriage. There are no known familial cases of similar disease, and the parents are non-consanguineous. The patient was born full term with a birth weight of 4000 g and a length of 53 cm. Apgar scores were 8 and 9. The perinatal period was unremarkable.

At 5 months of age, the patient developed pruritus and jaundice. Laboratory testing revealed hyperbilirubinemia, with total bilirubin reaching 190 μmol/L and direct bilirubin 160 μmol/L, without signs of cytolysis. Liver biopsy demonstrated features of giant cell hepatitis with fibrosis (Ishak stage 3, grade 7). A follow-up biopsy 1 year later revealed chronic hepatitis with porto-central fibrosis and progression to cirrhosis. Concomitantly, cytomegalovirus infection (CMV) was diagnosed: IgM 0.2, IgG 56, avidity 82%, and CMV DNA detected by PCR.

At the age of 1 year, during a planned hospitalization, liver biochemistry revealed the following: alanine aminotransferase (ALT) 72 U/L, aspartate aminotransferase (AST) 80 U/L, alkaline phosphatase (ALP) 352 U/L, GGT27 U/L, total bilirubin 41 μmol/L, direct bilirubin 32.5 μmol/L (78% of total), total protein 65 g/L, glucose 4.06 mmol/L, cholesterol 2.55 mmol/L, and albumin 37.36 g/L. The patient was followed with a diagnosis of chronic hepatitis, presumably of CMV etiology. From 1.5 years of age, liver function tests were monitored regularly, and transaminase and bilirubin levels remained within normal ranges. At age 3, a transient episode of hyperbilirubinemia (total 43 μmol/L, direct 16 μmol/L) resolved spontaneously. From that point until age 14, he was monitored annually by a gastroenterologist and no evidence of cytolysis or cholestasis.

At age 14, following an episode of acute respiratory viral infection, the patient experienced recurrence of pruritus, jaundice, pale stools, dark urine, and weight loss. Biochemical testing showed a progressive increase in bilirubin levels (92, 133, 270, and 355 μmol/L), predominantly direct (81, 113, 221, and 292 μmol/L). Transaminase levels were mildly elevated (ALT 41 U/L, AST 49 U/L); GGT remained normal (27 U/L), ALP was 339 U/L, international normalized ratio (INR) was 1.2, and bile salts were elevated to 147 μmol/L. Abdominal ultrasound revealed hepatomegaly, cholecysto cholangitis, enlargement of the pancreatic head and splenomegaly. Magnetic resonance imaging (MRI) demonstrated hepatosplenomegaly, hypoplasia of the gallbladder, and a small cyst in the right kidney. Liver biopsy showed morphological features of fibrosis with a cholestatic component (F4 according to METAVIR). Administration of hepatoprotective therapy with ursodeoxycholic acid resulted in clinical improvement, with normalization of liver function tests and a decrease in bilirubin levels (total 35 μmol/L, direct 17.3 μmol/L).

No specific drug–disease interactions have been reported in patients with *USP53*-related cholestasis, and ursodeoxycholic acid is generally considered safe and well tolerated in cholestatic liver disease.

During the current hospitalization, viral and autoimmune hepatitis, alpha-1 antitrypsin deficiency, and Wilson’s disease were excluded. Ultrasound revealed hepatosplenomegaly, diffuse parenchymal changes in the liver, and signs of portal hypertension. Neurological consultation established a diagnosis of Adie-Holmes syndrome. Audiological testing showed normal hearing.

The patient was discharged with the following diagnoses: cryptogenic (congenital) cholestatic hepatitis, liver cirrhosis (F2, METAVIR), and portal hypertension syndrome.

On physical examination, the patient appeared asthenic. His height was 186 cm (standard deviation score (SDS +1.92), and his weight was 61 kg (SDS +0.01). Kyphosis was noted. Scleral icterus was present.

**FIGURE 1:**
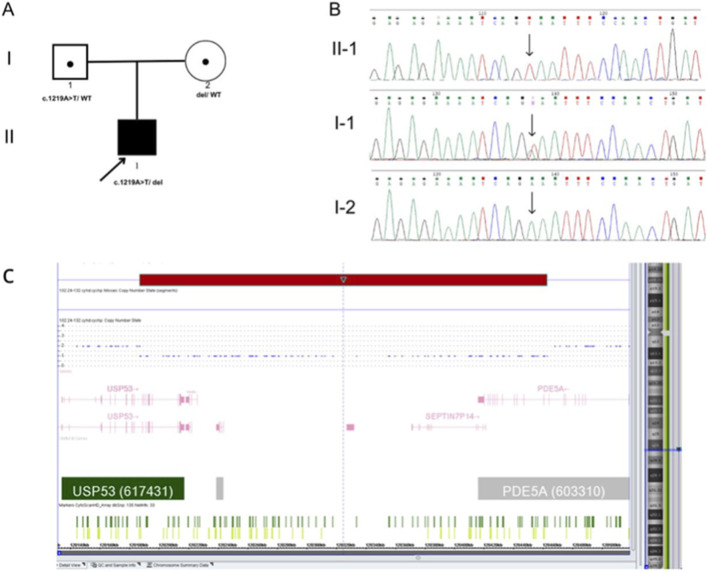
The pedigree and genetic testing of the proband and the results of sanger sequencing. **(A)** Pedigree showing the affected proband and their unaffected parents, both of whom are heterozygous carriers. **(B)** Sanger sequencing results demonstrating a hemizygous truncating *USP53* variant in the proband inherited from the father, in whom it is present in the heterozygous state. **(C)** CMA showing a maternally inherited heterozygous 275 kb deletion at 4q26 involving exons 13–19 of *USP53* and the terminal exon of *PDE5A*.

WES identified a previously unreported variant in exon 14 of the *USP53* gene in a homozygous or hemizygous state: NM_001371395.1:c.1219A>T, resulting in a premature stop codon p. (Lys407Ter) in exon 14 as it is shown in Figures 1A,B (Robinson et al., 2022). This variant was inherited from the father and was not present in the Genome Aggregation Database (gnomAD v4.1.0). Coverage analysis of the sequencing data suggested the presence of a heterozygous deletion in *USP53* on chromosome 4.

To confirm this deletion, chromosomal microarray analysis (CMA) was performed and revealed a microdeletion at 4q26 (GRCh38): chr4:119,265,361–119,541,176 (275 kb). This deletion encompasses exons 13–19 of the *USP53* gene. It was maternally inherited, as confirmed by Sanger sequencing and pedigree analysis, as shown in Figure 1C. Large deletions removing the last exons, including the natural stop codon, are known to trigger non-stop decay (NSD) rather than nonsense-mediated decay (NMD), as the resulting transcript lacks a proper termination signal. In our case, the deletion removes the terminal exons without introducing a premature termination codon (PTC) upstream, resulting in an mRNA that lacks an in-frame stop codon. During translation, ribosomes read through into the 3′untranslated region (UTR) or beyond and stall at the mRNA’s end, which activates the NSD surveillance pathway to degrade such “non-stop” mRNAs and prevent the production of aberrant proteins. Importantly, this deletion also affects the *USP53* catalytic domain, which is essential for its deubiquitinase activity. To date, this is the only CNV in the *USP53* gene reported in the literature.

Notably, the deletion also includes the terminal exon 21 of the adjacent *PDE5A* gene, which encodes a cGMP-specific phosphodiesterase involved in smooth muscle regulation and intracellular signal transduction. Although *PDE5A* is broadly expressed and pharmacologically relevant, it has not been conclusively associated with any monogenic human disorder.

According to the gnomAD v4.1.0 database, *PDE5A* has a low pLI score of 0.01 and a moderately reduced observed/expected ratio for loss-of-function variants (o/e = 0.49, 95% CI: 0.35–0.70), indicating tolerance to heterozygous loss-of-function events. This suggests that *PDE5A* is unlikely to cause autosomal dominant disease when disrupted in a single allele.

According to the ACMG/AMP guidelines (Richards et al., 2015), the c.1219A>T variant in *USP53* meets criteria PVS1 and PM2. Based on this evidence, it is classified as a pathogenic. This variant has been submitted to ClinVar as pathogenic by the Center for Precision Genome Editing and Genetic Technologies for Biomedicine, Pirogov Russian National Research Medical University on 13 April 2025 (ClinVar accession: RCV005002072.2; https://www.ncbi.nlm.nih.gov/clinvar/RCV005002072.2).

A maternally inherited heterozygous 275 kb deletion at 4q26 (GRCh38): chr4:119,265,361–119,541,176, affecting the second *USP53* allele meets criteria PVS1 and PM2 and is classified as likely pathogenic. This deletion was also submitted to ClinVar by the Research Centre for Medical Genetics on 19 July 2025 (ClinVar accession: VCV004057271.1, https://www.ncbi.nlm.nih.gov/clinvar/variation/4057271/).

Together, two variants—*USP53* c.1219A>T and the 4q26 deletion—located on opposite alleles and consistent with autosomal recessive inheritance, are considered the cause of the disease and support the diagnosis of PFIC 7.

## Discussion

USP53 has been predicted to be a catalytically inactive deubiquitinase based on its sequence similarity to other members of the USP family. Structural studies have demonstrated that USP53 lacks a critical histidine residue within the catalytic triad, classifying it as a non-protease homolog within the USP family. Despite this predicted inactivity, experimental evidence indicates that USP53 performs important non-enzymatic functions. Studies in mouse models have revealed that USP53 regulates metabolism, bone remodeling, liver function, and hearing. In particular, USP53 contributes to the maintenance of tight junction integrity through interactions with TJP1 and TJP2, and its loss leads to canalicular membrane instability and cholestasis. Recent work by Xia et al. further summarized emerging evidence that, although catalytically inactive, USP53 exerts significant physiological and pathological roles across multiple tissues, acting as a novel regulatory protein and a potential diagnostic and therapeutic target in metabolic, skeletal, hepatic, and neoplastic disorders (Xia et al., 2024).

In this study we describe a patient with compound heterozygous *USP53* variants, including a novel nonsense variant (p.Lys407Ter) and a large deletion encompassing exons 13–19. Despite the presumed pathogenic impact of these disruptive variants, the patient demonstrated a mild BRIC-like phenotype, characterized by intermittent episodes of pruritus and hyperbilirubinemia with stable liver function and no progression to hepatic failure. Histological evaluation revealed fibrosis, consistent with PFIC, but without inflammation or hepatocellular damage. Although CMV infection was detected serologically and by PCR, the serological profile indicated a past infection, and the liver histology lacked features typical of CMV hepatitis. Therefore, CMV was considered an incidental finding with no significant contribution to the liver pathology.

**FIGURE 2:**
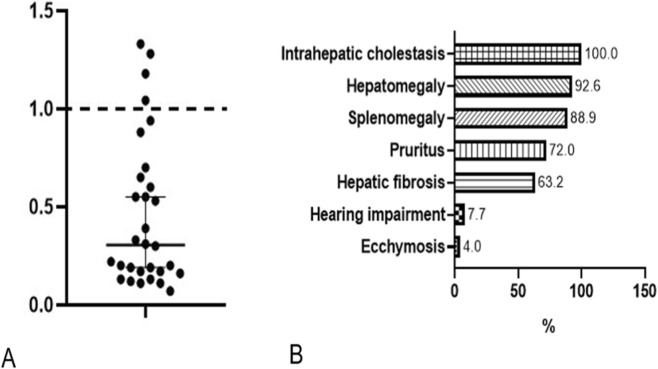
GGT ratio in all published patients **(A)**. Frequency of phenotypic abnormalities, HPO terminology **(B)**.

Given the unexpectedly benign course in this case, we conducted a comprehensive literature review to contextualize the variability in genotype and phenotype of *USP53*-related cholestasis. The review was performed in accordance with PRISMA guidelines in a simplified form. The PubMed database was systematically searched using the keywords *“USP53”* and *“cholestasis”*, covering all years available up to December 2024. Titles and abstracts were screened to identify reports describing human patients with *pathogenic or likely pathogenic* variants in *USP53*. Duplicates, review articles, experimental or animal studies, and papers lacking clinical or genetic data were excluded. After applying these criteria, 39 published cases were included for analysis. Clinically, cholestasis and hepatomegaly were nearly universal features across cases as it is shown in Figure 2B. In terms of disease course, BRIC-like episodes were described in 17.7% (6/34) of patients, while intermittent pruritus with mildly elevated transaminases was noted in 26.5% (9/34). The majority (76.5%, 26/34) maintained normal or only mildly elevated liver enzyme levels during follow-up. Only one patient (2.9%) progressed to hepatic failure and another died 2 years after liver transplantation performed for intractable pruritus. Histological findings in *USP53*-related cholestasis commonly show canalicular cholestasis, ductular proliferation and fibrosis without inflammation. Detailed clinical outcomes and patient-level data are provided in the Table 1.

**TABLE 1:** Summary of clinical, biochemical, histological, imaging, genetic, and outcome data from 39 individuals with biallelic *USP53* variants reported in the literature, including the current case. Data were extracted from 12 published sources and grouped to illustrate the phenotypic spectrum ranging from BRIC to PFIC-like disease.

References	N	Age of onset (Me, mo)	Gender	Main clinical features	Liver biopsy findings	BRIC
Our case	1	12	m	C, P, hepatomegaly, S, Adie-Holmes syndrome	HF	1/1
P et al. (2021)	1	0,33	m	C, P, H in all	HF Ductular proliferation, canalicular cholestasis, giant cell transformation	0/1
Alhebbi et al. (2020)	8 from 8 unrelated families	(13,5 [5.5; 105])	4 m/4f	C, P, H, S in all, hearing impairment in 2 patients. 1 died	HF (2/8), ductular proliferation, canalicular cholestasis (2/8)	2/8
Bull et al. (2020)	7 from 5 unrelated families	(5 [2; 84])	4 m/3f	C, H, S in all	HF (4/7), ductular proliferation, canalicular cholestasis (4)	0/7
Zhang et al. (2020)	7 from 7 unrelated families	(5 [0,1])	4 m/3 f	C, hearing impairment in 1 patients	ductular proliferation in all	0/7
Samanta et al. (2024)	3 from 3 unrelated families	(8 [6; 16])	1 m/2f	C, P, H, S in all	HF in all, ductular proliferation in 2 patients	0/3
Berberoğlu Ateş et al. (2023)	1	6	1 m	C, P, H, S in all	Ductular proliferation, canalicular cholestasis	1/1
Ahn et al. (2023)	1	252	1 m	C	Giant cell transformation	1/1
Shatokhina et al. (2021)	1	0	1 m	C, P, H, S in all Other findings: multiple hepatic hemangiomas	HF	1/1
Gezdirici et al. (2022)	1	4	1 f	C, P, H, S in all	HF in all, ductular proliferation	0/1
Cheema et al. (2023)	2	(54 [36; 72])	2 f	C, P, H, S in all	N/D	0/2
Zheng et al. (2023)	1	7	1 f	C, E	N/D	0/1
Cheema et al. (2020)	5	5, N/D for all patients	N/D	C	N/D	N/D/

C, cholestasis; E, ecchymosis; P, pruritus; H, hepatomegaly; S, splenomegaly; HF, hepatic fibrosis; N/D, no data; m, male; f, female; mo, month; y, year; N, number of patients.

PFIC7 is typically associated with normal or mildly elevated GGT, a key biochemical marker. To account for age- and sex-specific reference ranges, we calculated a GGT ratio for each individual by dividing the measured GGT value by the upper limit of normal for that person’s age and sex. Among 30 patients with *USP53*-related disease, 26 had GGT ratios ≤1.0 (normal), while 4 showed mildly elevated ratios not exceeding 1.5. These findings support the consistent low- or normal-GGT biochemical profile characteristic of this disorder (Figure 2A).

**FIGURE 3:**
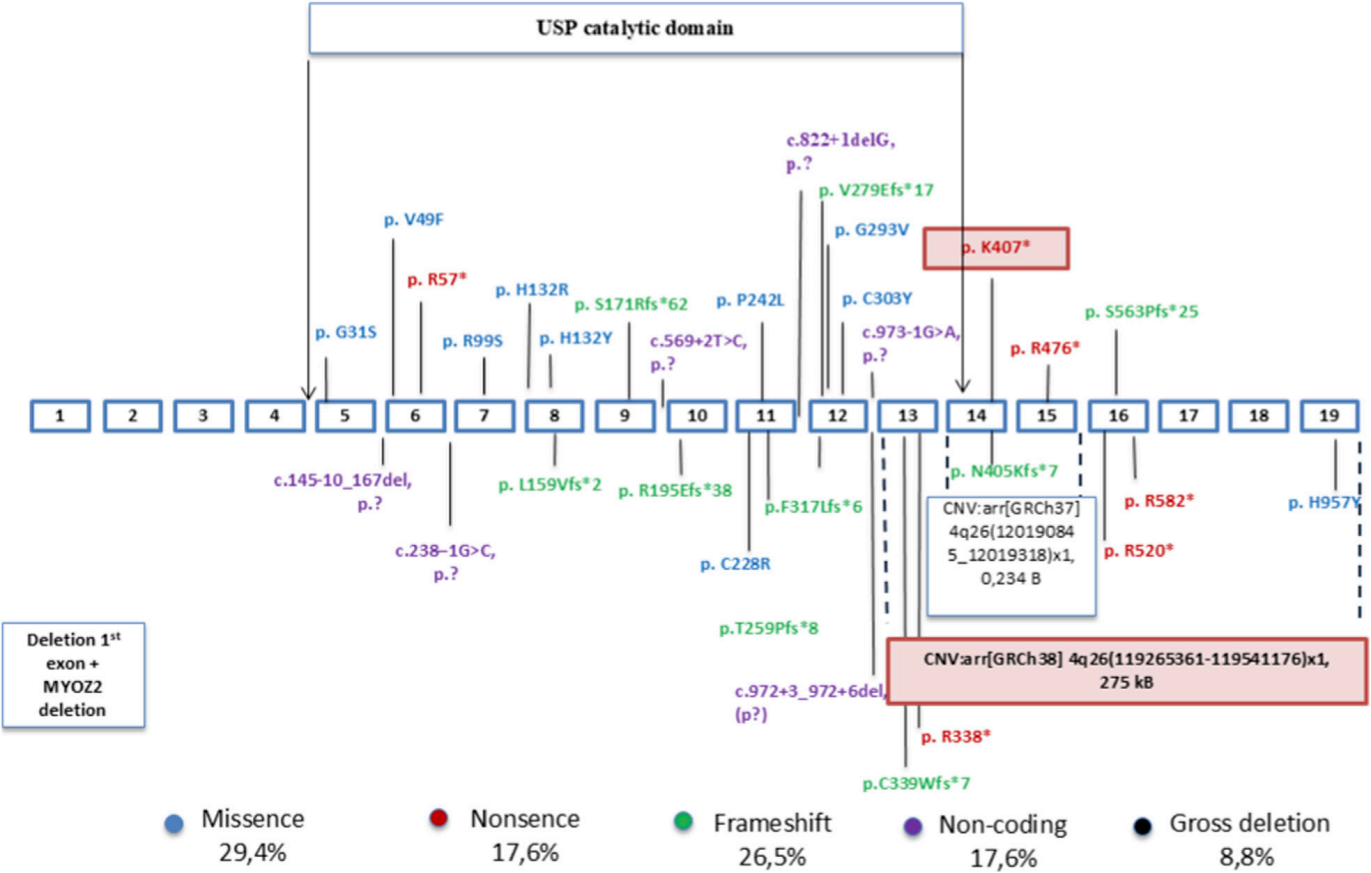
Previously reported and novel protein-level variants in the *USP53* gene. Legend. Schematic representation of 32 all previously reported pathogenic and likely pathogenic protein-level variants in the *USP53* gene, along with two novel variants identified in the present case: a nonsense variant in exon 14 (p.Lys407*) and a heterozygous deletion encompassing exons 13–19. The exonic structure and variant positions are shown relative to the USP catalytic domain.

The mutation spectrum highlights that most reported variants are loss-of-function, including frameshift, nonsense, splicing defects, and gross deletions as shown in Figure 3. Missense variants were less common. Notably, gross deletions like the one identified in our case remain rare, documented in only a few reports to date.

Although initially classified as PFIC7 in OMIM (2019), our review of 39 cases reveals that the majority exhibit a phenotype more typical of BRIC. Although periportal fibrosis was identified on liver biopsy, the overall clinical course—marked by episodic cholestasis, long asymptomatic intervals, and preserved hepatic function—remains consistent with a BRIC-like phenotype. These findings suggest that *USP53*-related disease may exist along a clinical spectrum, with histological features of PFIC but functional and clinical behavior resembling BRIC.

Despite carrying a gross deletion in *USP53*, our patient exhibited a BRIC-like phenotype with intermittent cholestasis and preserved liver function, supporting the notion that *USP53*-related disease spans a clinical spectrum, and even disruptive variants may present with mild courses.

A limitation of this study is the inclusion of a single patient, which restricts the generalizability of the findings. Nevertheless, the detailed clinical, genetic, and histological characterization contributes valuable information to the understanding of *USP53*-related cholestasis.

## Data Availability

The original contributions presented in the study are included in the article/supplementary material, further inquiries can be directed to the corresponding author.
